# Vitamin D enhances type I IFN signaling in COVID-19 patients

**DOI:** 10.1038/s41598-022-22307-9

**Published:** 2022-10-22

**Authors:** Shirin Hafezi, Fatemeh Saheb Sharif-Askari, Narjes Saheb Sharif-Askari, Hawra Ali Hussain Alsayed, Habiba Alsafar, Fatme Al Anouti, Qutayba Hamid, Rabih Halwani

**Affiliations:** 1 Research Institute of Medical & Health Sciences, University of Sharjah, Sharjah, United Arab Emirates; 2 Pharmacy Department, Dubai Health Authority, Dubai, United Arab Emirates; 3 Department of Biomedical Engineering, College of Engineering, Khalifa University of Science and Technology, Abu Dhabi, United Arab Emirates; 4 Center for Biotechnology, Khalifa University of Science and Technology, Abu Dhabi, United Arab Emirates; 5 Department of Genetics and Molecular Biology, College of Medicine and Health Sciences, Khalifa University of Science and Technology, Abu Dhabi, United Arab Emirates; 6 Department of Health Sciences, Zayed University, Abu Dhabi, United Arab Emirates; 7 Department of Clinical Sciences, College of Medicine, University of Sharjah, Sharjah, United Arab Emirates; 8 Meakins-Christie Laboratories, Research Institute of the McGill University Health Center, Montreal, QC Canada; 9 Prince Abdullah Ben Khaled Celiac Disease Chair, Department of Pediatrics, Faculty of Medicine, King Saud University, Riyadh, Saudi Arabia

**Keywords:** Inflammation, Innate immunity, Cell signalling

## Abstract

The ability of Vitamin D (VitD) to modulate antiviral responses through induction of antimicrobial peptide is well established. However, the effect of VitD on host responses to SARS-CoV-2 is not well investigated. We here report the ability of VitD to enhance host IFN-alpha/beta (a/β) signaling both in vitro and among severe COVID-19 patients treated with VitD. Blood and saliva specimens were obtained from severe COVID-19 patients treated (43 patients), or not (37 patients), with vitD, during their stay in intensive care unit. Patients were followed up to 29 days following admission, and patient survival outcomes were collected. Higher activity levels of RIG-1/MDA-5 and JAK-STAT signaling pathways were observed with significantly higher gene and protein levels of antiviral interferon stimulating genes (ISGs) such as MX-1 and ISG-15; both in vitro, following treatment of PBMCs with vitD, and in whole blood and saliva specimens of VitD treated patients. Moreover, VitD treated patients had lower risk of all-cause mortality by day 29 compared to untreated patients (adjusted hazard ratio, 0.37, 95% confidence interval of 0.14–0.94; P = 0.038). The herein uncovered regulatory role of VitD on type I IFNs suggests the importance of insuring a normal level of VitD for the prevention and probably treatment of SARS-CoV-2 infection. Additional mechanistic studies, however, are needed to fully elucidate the antiviral effects of VitD particularly in the setting of COVID-19 infection.

## Introduction

Innate immunity is critical for controlling SARS-CoV-2 infection; and patients with dysregulated innate immunity are prone to development of severe disease^1^. Type I interferons (IFN-α, and β) represent key elements of antiviral innate immunity. Several reports have shown that efficient induction of IFN-α/β signaling and the resultant interferon stimulating genes (ISGs) are essential for the control and resolution of SARS-CoV-2 infection^2^.

SARS-CoV-2 is recognized in the cytosol of human epithelial cells by single-stranded (ss)RNA cytosolic sensing proteins (RIG-1 and MDA5)^3,4^. This will then lead to the downstream activation of interferon regulatory factors (IRF)3, or IRF7, and rapid production of IFN α/β cytokines, which exhibit key antiviral activity, thereby limiting viral proliferation and spreading^5^. IFN α/β cytokines bind to a dimeric receptor composed of IFNAR1 and IFNAR2 subunits; consequently, triggering the formation of transcription complex, IFN-stimulated gene factor 3 (ISGF3). ISGF3, which consists of phosphorylated signal transducer and activator of transcription STAT(1), STAT2 and IRF9, migrates to the nucleus, binds to interferon-stimulated response elements (ISREs), and activates transcription of anti-viral ISGs^6–8^. Collectively, the efficient induction of IFN-α/β signaling and ISGs in virus-infected cells is fundamental for the antiviral response of a host. As such, studies have reported that COVID-19 patients with a genetic defect in the production of IFN-α/β cytokines or having autoantibodies which neutralize these cytokines, could suffer from worse clinical outcomes^1,9,10^.

To date, there is no known effective antiviral agent approved for the management of COVID-19 disease. Several reports have shown the effectiveness of interferon-based therapy (IFNβ1a or IFNβ1b) against SARS-CoV-2 infection in different settings of hospitalized COVID-19 patients^11–13^. Moreover, interferon therapy has been shown to reduce viral load and improve lung pathology in a nonhuman primate model of coronavirus infection^14^. Thus, the search for therapeutic strategies that can stimulate IFN levels and prevent SARS-CoV-2 infection is becoming more important.

Vitamin D (VitD) has long been recognized as an essential vitamin for the skeletal system. Further evidence suggests that it also plays a major role in regulating the immune system, including immunity to viral infections^15,16^. Epidemiological studies have shown that VitD deficiency may confer increased risk of influenza and COVID-19 severity^17^, while supplementation on the other hand might prevent progression of COVID-19 or death^18–20^. in vitro studies also supported the fact that VitD has direct anti-viral effects and linked that to VitD's ability to up-regulate the anti-microbial peptides, including cathelicidin LL-37 and human beta defensin 2^15,21,22^. Here, we have shown, in vitro and in the setting of COVID-19 hospitalized patients, that VitD's anti-viral mechanism can be linked to its ability to increase host type I IFN immunity by increasing the activities of RIG-1/MDA-5 signaling, JAK-STAT pathway, and the resultant IFN α/β signaling and ISGs production.

## Methods

### In-Silico dataset

We have used transcriptomic datasets publicly available at National Center for Biotechnology Information Gene Expression Omnibus (NCBI GEO, http://www.ncbi.nlm.nih.gov/geo) and the European Bioinformatics Institute (EMBL-EBI, https://www.ebi.ac.uk). Studies (RNA-sequencing platforms) included were bronchial epithelial cells cultured and treated with or without calcitriol (GSE106885), poly I:C (GSE106885), IFNα (GSE148829), or SARS-CoV-2 (GSE147507). Reactome interferon alpha/beta signaling pathway (R-HSA-909733) and Human ISG library consisting of 524 genes^23,24^ were used to screen for IFN/ISGs levels in treated airway epithelial cells. Details of the datasets used are presented in supplementary Table S1.

### COVID-19 patients’ cohort

We obtained blood and saliva specimens from 80 patients with severe COVID-19-related pneumonia with confirmed SARS-CoV-2 infection by PCR, and hospitalized at intensive care unit at the Rashid Hospital in Dubai between September 2020, and January 2021. The COVID-19 severity status was defined as pneumonia requiring high-flow oxygen therapy or non-invasive ventilation^25^. At the time of the study, 43 of these patients were treated with VitD (cholecalciferol) administrated as 50,000 IU weekly for 2–3 weeks. The allocation of patients to VitD was based on national treatment protocols that recommended addition of 50,000 units of VitD once weekly, and based on the physician judgment^25^. The rest of the patients (n = 37) did not receive VitD. Laboratory and clinical data at admission is listed in Table 1, with exception for serum 25-hydroxyvitamin D [25(OH)D] level as it was not routinely required per national clinical management protocols of COVID-19^25^. The selected patients were adjusted for all the demographics, comorbidities, and common laboratory inflammatory markers of COVID-19 severity, and the use of any kind of interferon-based therapy such as IFNβ1a or IFNβ1b (Table 1). All the specimens were collected as a part of patient routine clinical care. Saliva was collected by suction from intubated patients. The ethical approval for this study was obtained from the Dubai Scientific Research Ethics Committee (DSREC), Dubai Health Authority at Rashid Hospital (DSREC-12/2020_02). Written informed consent was obtained from all the study participants. All methods were performed in accordance with the relevant guidelines (Declaration of Helsinki and the Belmont Report) and regulations (DSREC rules). Precautions recommended by CDC for safe collection, handling and testing of biological fluids were followed^26^.

**Table 1:** Baseline clinical characteristics of the study population.

Variables	VitD treated patients (n = 43)	Untreated patients (n = 37)	p-value
Age, years	60 (38–93)	54 (21–84)	0.078
Male sex	36 (84)	27 (73)	0.281
BMI	28 (19–42)	28 (17–41)	0.644
**Underlying comorbidities**			
Diabetes	25 (58)	15 (40)	0.178
Cerebrovascular disease	27 (63)	17 (46)	0.177
Obesity	14 (32)	12 (32)	0.990
**Baseline laboratory data (normal range)**			
White cell count (3.9–11.10 × 10^9^ per L)	6.5 (4.8–8.5)	7.8 (6.4–9.9)	0.344
C-reactive protein (1.0–3.0 mg/L)	123.7 (105–216)	82.7 (37–174)	0.127
D-Dimer (0–0.5 μ/mL)	1.24 (0.64–1.96)	1.17 (0.66–2.31)	0.583
Ferritin (10–204 ng/mL)	878.4 (432–1579)	480.6 (261–1193)	0.296
**COVID-19 supportive medication**			
Tocilizumab	24 (59)	16 (43)	0.370
IFN-α/β	6 (14)	7 (20)	0.562

Data are n (%) or median (IQR).

*BMI* body mass index, *CRP* C-reactive protein.

### In vitro treatment

Human peripheral blood mononuclear cells (PBMCs) were isolated from the peripheral blood of 3 healthy controls using Ficoll-Paque, and were resuspended in complete RPMI-1640 media. Furthermore, the cells were treated, or not, with 50 nM calcitriol (Sigma-Aldrich, USA) and/or 1 μg/ml of IFNα (Myltenic Biotec, USA) for 8 h. Then, the total RNA and proteins were isolated from these cells in order to perform RT-qPCR and western blot assay, respectively.

### Protein expression by western blot

Whole blood and saliva samples were pelleted by centrifugation at 14,000*g* for 20 min at 4 °C. The protein concentrations were measured using the BCA protein assay reagent kit (Thermo-Scientific Pierce BCA Protein Assay Kit). Cells were lysed using 10X RIPA Buffer (Abcam) after supplementation with 1 × Protease Inhibitor Cocktail (Sigma-Aldrich, USA) and 1 mM phenylmethylsulfonyl fluoride (Sigma-Aldrich, USA). Fifteen micrograms total proteins were separated using 10% gels. The proteins were transferred onto a nitrocellulose membrane (Bio-Rad), blocked in skimmed milk for 1 h at room temperature, incubated overnight at 4 °C with antibodies specific to VDR (cat#12,550), p-STAT1 (cat#9167), STAT1 (cat#14,994), p-STAT2 (cat#88,410), STAT2 (cat#72,604), p-JAK1 (cat#74,129), JAK1 (cat#29,261), p-IRF3 (cat#29,047), IRF3 (cat#11,904), RIG-1 (cat#3743), MX-1 (cat#37,849), and ISG-15 (cat#2758). β-Actin (cat#8457) was used as loading controls. All the antibodies were purchased from Cell Signaling Technology, USA. The blots were developed using the Clarity Western ECL Substrate (Bio-Rad) in the ChemiDoc Touch Gel Imaging System (Bio-Rad). Image Lab software (Bio-Rad) was used to detect and quantify the protein bands.

### IFNα enzyme-linked immunosorbent assay

IFNα cytokine concentrations were determined in whole blood samples using commercially available human ELISA kit (Human Interferon alpha 1 ELISA Kit (ab213479), Abcam, USA). Assays were preformed following the manufacturer’s instructions. All samples were measured in duplicates.

### Gene expression assay using qRT-PCR

Total RNA from PBMCs, whole blood, and saliva samples was isolated using Trizol reagent according to the manufacturer’s instructions (Invitrogen, Carlsbad, CA)^27^. Complementary cDNA was synthesized from 1 μg of RNA using the High-Capacity cDNA Reverse Transcription Kit (Applied Biosystems) according to the manufacturer’s protocol. For cDNA amplification, 5 × Hot FirePol EvaGreen qRT-PCR SuperMix (Solis Biodyne, USA) was used, and qRT-PCR was performed in QuantStudio 3 Real-Time PCR System (Applied Biosystems)^28^. Primer sequences for MDA-5 (IFIH1), RIG-1 (DDX58), IRF3, IRF9, MX-1, ISG-15, and 18s used in qRT-PCR are deposited in Supplementary Table S2. Gene expression was analyzed using the Comparative Ct (ΔΔCt) method upon normalization to the reference gene 18s rRNA^29^.

### Analysis procedures

The raw Affymetrix data was normalized, and log transformed. Microarray data (CEL files) were pre-processed with Robust Multi-Array Average (RMA) technique using R software^30^. The probe set ID with the largest IQR of expression values were selected to represent the gene. For RNA-seq study, the data was pre-processed using the Bioconductor package *limma-voom*^31^. The fold change of differential expressed genes were carried out using *Limma* Bioconductor package^32,33^.

Moreover, association of VitD supplementation with mortality was evaluated using Cox proportional hazards regression model adjusted for patient’s demographics factors (age, gender, and body mass index), comorbidities (diabetes mellitus), and COVID-19 related severity serum marker (D-dimer and CRP). Kaplan–Meier survival curve was then constructed to show cumulative survival over the 29-day period. Statistical analysis was performed using SPSS (version 26.0), R software (version 3.6.1) and PRISM (version 8). All tests were two-tailed and a P value of less than 0.05 was considered statistically significant.

## Results

### VitD enhances IFN α/β signaling in vitro

**Figure 1:**
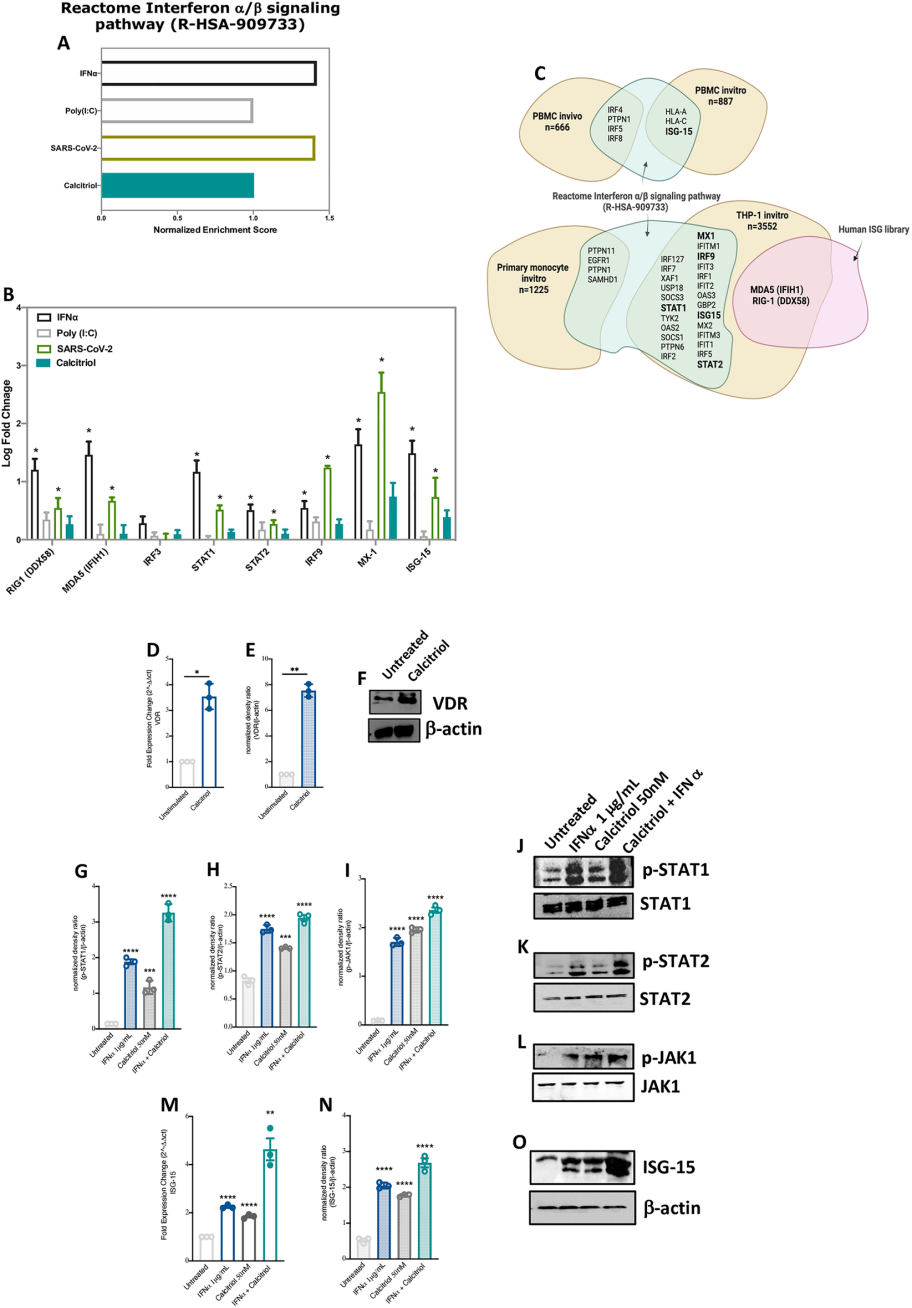
Increased IFNα/β pathway signaling and higher expression of ISGs in human airway epithelial cells and PBMCs following VitD treatment. (**A**) Enrichment of IFNα/β pathway in IFNα, poly I:C, SARS-CoV-2, and VitD treated human airway epithelial cells (HAECs). Normalized enrichment Score (NES) of IFNα (1.41), poly I:C (1.00), SARS-CoV-2 (1.40), and VitD (1.005) treated HAECs. (**B**) The gene expression levels of RIG-1, MDA-5, IRF3, IRF9, MX-1, and ISG-15 in IFNα, poly I:C, SARS-CoV-2, and VitD treated HAECs treated cells. (**C**) Overlays of genes in Reactome Interferon α/β signaling pathway (R-HSA-909733) and VitD target gene lists. For IFNα, the RNAseq data was obtained from 3 replicates of IFNα-treated BEAS-2B cells using GEO: GSE148829 dataset. For poly I:C or VitD, the RNAseq data was obtained from 3 replicates of poly I:C or calcitriol (VitD) treated HAECs using GEO: GSE106885 dataset, while for SARS-CoV-2, the RNAseq data was obtained from 3 replicates of SARS-CoV-2 infected HAECs were extracted from GEO: GSE147507 dataset. Results are presented as log fold change ± SE of gene expression between cases and controls. (**D**–**F**) The mRNA and protein levels of VDR in VitD (50 nM of calcitriol for 8 h) treated PBMCs. (**G**–**L**) Protein levels of p-STAT1, p-STAT2 and p-JAK1 in VitD and/or IFN**α** (50 nM of calcitriol and/or 1 μg/ml of IFN**α** for 8 h) treated PBMCs. (**M**–**O**) The mRNA and protein levels of ISG-15 in VitD and/or IFN**α** treated PBMCs. PBMCs were isolated from peripheral blood of healthy donors (n = 3). Statistic test: comparison was done using unpaired t-test or Mann–Whitney U test, depending on the skewness of the data. *P < 0.05, **P < 0.01, ***P < 0.001, ****P < 0.0001.

Using publicly available transcriptomic datasets, we evaluated the activity of reactome type I IFN signaling pathway (R-HSA-909733) following treatment of human airway epithelial cells (HAECs) with VitD (calcitriol), poly I:C (mimicing viral infections), IFNα, as well as following SARS-CoV-2 infection. Interestingly we noticed that IFN α/β signaling pathway was enriched or activated in vitro following VitD treatment as it was the case for IFNα or poly I:C treated cells, and SARS-CoV-2 infected cells. (Fig. 1A, Normalized enrichment Score (NES) of IFN α/β signaling pathway was 1.005 for VitD, 1.41 for IFNα, 1.00 for poly I:C, and 1.40 for SARS-CoV-2).

In line with this, the expression of RNA cytosolic sensing proteins, such as RIG-1 (DDX58) and MDA5 (IFIH1); interferon regulatory proteins, such as IRF3 and IRF9; and interferon stimulating genes, such as MX-1 and ISG-15 were elevated in HAECs following VitD treatment similar to IFNα and poly I:C treatments, as well as SARS-CoV-2 infection (Fig. 1B). These results suggest that VitDIFN-mediated antiviral responses could be through activation of RIG-1/MDA-5 signaling, JAK-STAT pathway, and the resultant ISGs production.

To further confirm the effect of VitD on these genes in lung cells, we have used four different lists of well-known VitD target genes that were obtained from two cell types (PBMCs or monocytes) following treatment with VitD, either in vivo or in vitro^34–37^. One dataset was for PBMCs of individuals who were given a bolus of vitamin D3 (2000 µg or 80,000 IU)^34^; another for PBMCs that were isolated from 12 healthy individuals and were treated in vitro with 10 nM of 1,25(OH)_2_D_3_ (calcitriol)^35^; a third dataset was for primary monocytes that were isolated from PBMCs of 85 individuals and then treated in vitro with 100 nM of calcitriol^36^, and the fourth dataset was for THP-1 cells which had been treated in vitro with 100 nM of calcitriol^37^. Interestingly, several reactome IFN α/β signaling genes that were upregulated following treatment of lung cells with VitD (RIG-1, MDA5, STAT1, STAT2, IRF9, MX-1, ISG-15) overlapped with those already known to respond to VitD in PBMCs or monocytes (Fig. 1C; Supplementary Table S3).

Moreover, we examined the regulatory effect of VitD on Type I IFNs by treating PBMCs isolated from healthy subjects with 50 nM VitD (calcitriol) and/or IFNα. We first examined the expression of VitD receptor (VDR) on those cells at baseline and following treatment with VitD. Of note, higher mRNA and protein expression levels of VDR were observed following VitD treatment (Fig. 1D–F, 3 log FoldChange (FC) increase of VDR mRNA level; P = 0.002). Moreover, the levels of phosphorylated (p)-STAT1, p-STAT2, and p-JAK1 were upregulated in PBMCs following treatment with VitD and/or IFNα (Fig. 1G–L, p < 0.0001; Supplementary Fig. S1). Consequently, higher gene and protein expression levels of ISGs such as ISG-15 were noticed in these treated cells (Fig. 1M–O, 2 FC increase of ISG-15 mRNA level, P < 0.000; Supplementary Fig. S1). This higher activity of JAK-STAT signaling and ISG expression level following treatment with VitD is indicative of increased IFN α/β signaling.

### Type I IFN signaling is enhanced in blood of VitD treated COVID-19 patients

Dysregulation of Type I IFNs is believed to be critical for the development of severe COVID-19 disease^10^. Hence, we investigated whether treatment of severe COVID-19 patients with VitD may enhance their Type I IFN signaling. For this purpose, we have measured the activity of RIG-1/MDA-5 and JAK-STAT signaling pathways in whole blood of severe COVID-19 patients treated, or not, with VitD (50,000 IU cholecalciferol once per week for 2–3 weeks) during their hospital stay. We included 80 COVID-19 patients during their stay at ICU. Out of these patients, 43 patients were treated with VitD, while 37 did not receive VitD treatment. There was no significant difference at baseline in demographics, comorbidities, and markers of COVID-19 severity among these two groups (Table 1).

**Figure 2:**
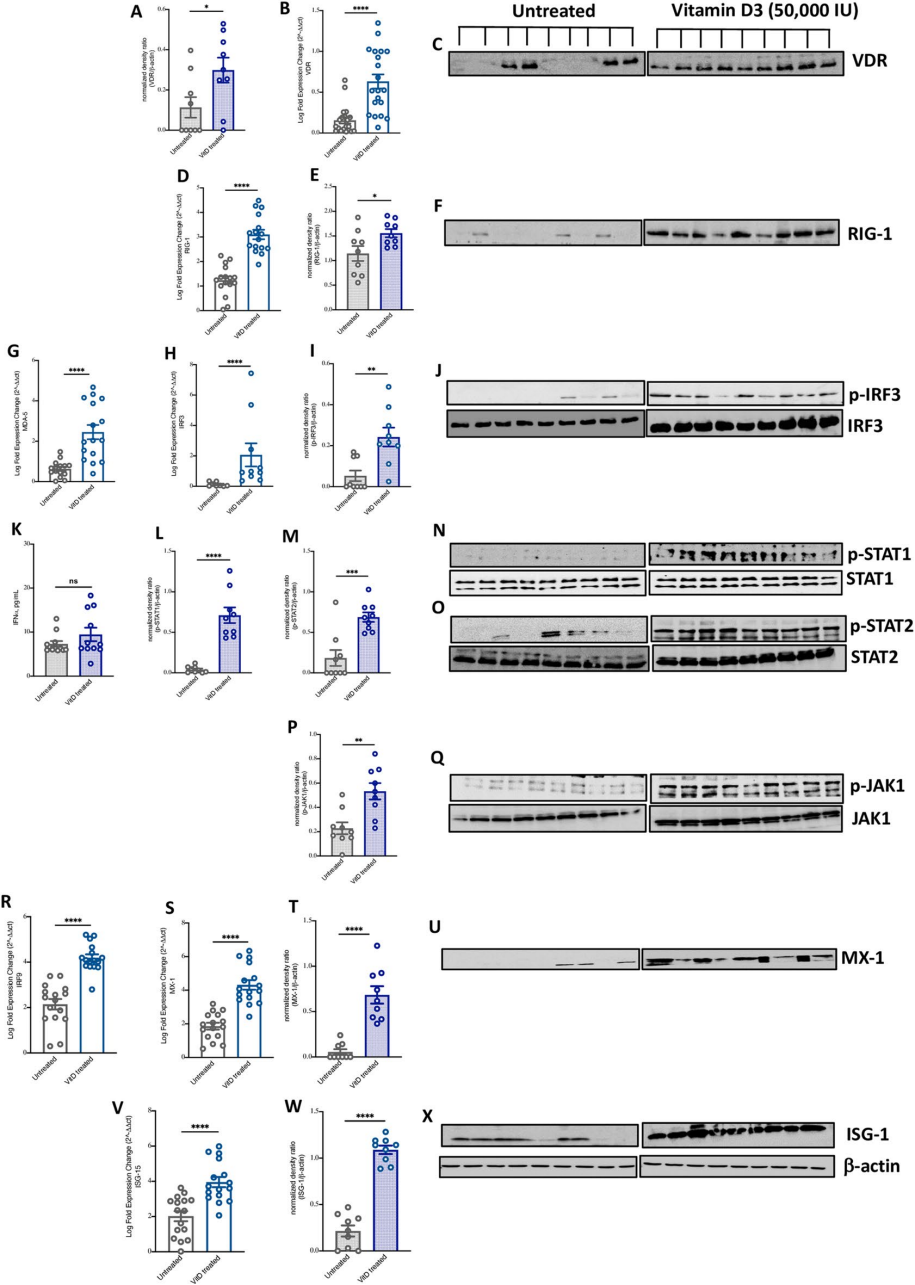
Increased IFNα/β signaling in blood of VitD treated COVID-19 patients. (**A**–**C**) The mRNA and protein levels of VDR in whole blood of VitD treated (cholecalciferol was administrated as 50,000 IU weekly) and untreated COVID-19 patients. (**D**–**J**) The mRNA and/or protein levels of RNA cytosolic sensing proteins, such as RIG-1, MDA5, and p-IRF3 in whole blood of VitD treated and untreated COVID-19 patients. (**K**) The plasma levels of IFN**α** in VitD treated and VitD untreated COVID-19 patients. (**L**–**Q**) Protein levels of p-STAT1, p-STAT2, and p-JAK1 in blood of VitD treated and VitD untreated COVID-19 patients. (**R**–**X**) The mRNA and/or protein levels of IRF3 and ISGs such as MX-1 and ISG-15 in blood of VitD treated and VitD untreated COVID-19 patients. The whole blood of VitD treated (n = 9; 6 males and 3 females) and untreated COVID-19 patients (n = 9; 6 males and 3 females) were randomly chosen from cohorts of VitD treated (n = 43; 36 males and 7 females) or untreated (n = 37; 27 males and 10 females) COVID-19 patients. Statistic test: comparison was done using unpaired t-test or Mann–Whitney U test, depending on the skewness of the data. *P < 0.05, **P < 0.01.

We first measured the expression level of VDR in the blood of these patients. Higher mRNA and protein levels of VDR was observed in blood of COVID-19 patients receiving VitD compared to those who did not (Fig. 2A–C, 0.5 log FC increase of VDR mRNA level, P < 0.0001). Interestingly, we observed an elevated mRNA and/or protein levels of RIG-1, MDA-5, and p-IRF3 in whole blood of treated compared to untreated patients (Fig. 2D–J 2 log FC increase of MDA-5 and RIG-1 mRNA levels; P < 0.0001; and 3.76 log FC increase of IRF3 mRNA, P = 0.03). As these events may lead to rapid production of IFN-α/β cytokines^5^, we further measured the levels of IFNα in the plasma of these patients using ELISA assay. An increase in plasma levels of IFNα following VitD was observed, although not to a significant level (Fig. 2K, mean 9.4 ± 2.2 vs mean 7.3 ± 5 pg/mL, of IFNα level in plasma of VitD treated vs untreated patients, P = 0.196).

Moreover, to confirm the regulatory effect of VitD on IFN α/β signaling pathway, we measured activity of JAK-STAT pathway in whole blood of our recruited severe COVID-19 patients. Interestingly, we observed an elevated level of phosphorylated p-STAT1, p-STAT2, and p-JAK1 in blood of VitD treated compared to non-treated COVID-19 patients (Fig. 2L–Q, P < 0.0001; Supplementary Fig. S2). Moreover, the mRNA and/or protein levels of IRF9 as well as ISGs such as MX-1 and ISG-15 were elevated following VitD treatment (Fig. 2R–X, around 2 log FC increase of IRF9, MX-1, and ISG-15 mRNA levels, P < 0.0001). This suggests that VitD treatment enhances the activity of type I IFN signaling resulting in better immunity against viral infections, specifically pertaining to SARS-CoV-2.

### VitD stimulated IFN α/β signaling is reflected in saliva of treated COVID-19 patients

Saliva fluid is believed to be a good source of non-invasive biomarkers for COVID-19 diagnosis and prognosis^38^. We hence decided to examine whether the observed effect of VitD on type I IFNs could be reflected in saliva fluid. This would, hence, suggest the potential use of saliva for measuring type I IFN activity following treatment with medications or vitamin supplementations. To do that, we measured the activity of RIG-1/MDA-5 and JAK-STAT pathways in the saliva samples obtained from severe COVID-19 patients treated, or not, with VitD.

**Figure 3:**
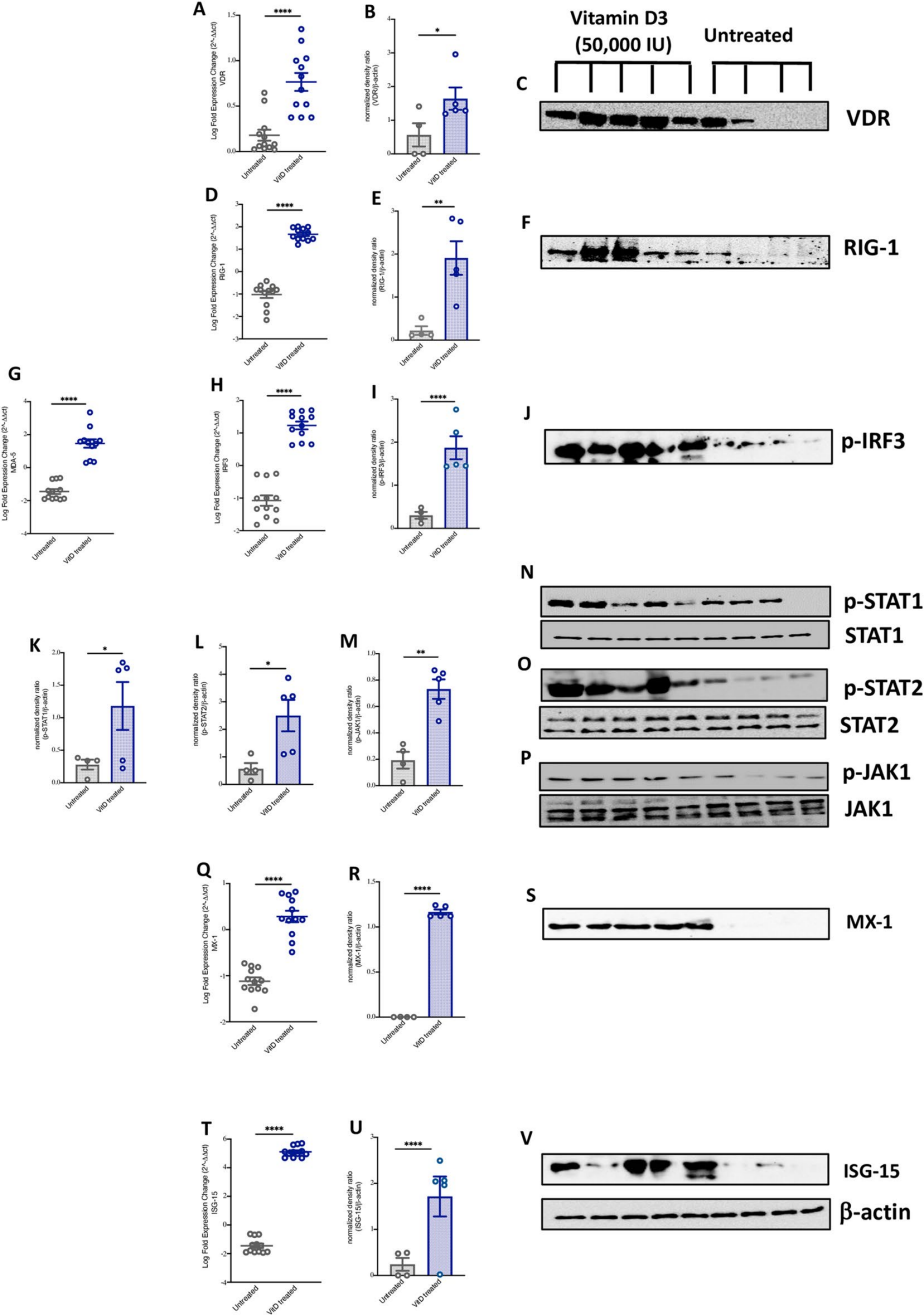
Increased IFNα/β signaling in saliva of VitD treated COVID-19 patients. (**A**–**C**) The mRNA and protein levels of VDR in saliva of VitD treated (cholecalciferol was administrated as 50,000 IU weekly) and untreated COVID-19 patients. (**D**–**J**) The mRNA levels of RNA cytosolic sensing proteins, such as RIG-1, MDA5, and p-IRF3 in saliva of VitD treated and untreated COVID-19 patients. (**K**–**P**) Protein levels of p-STAT1, p-STAT2, and p-JAK1 in saliva of VitD treated and VitD untreated COVID-19 patients. (**Q**–**V**) The mRNA levels of ISGs such as MX-1 and ISG-15 in saliva of VitD treated and VitD untreated COVID-19 patients. The saliva of VitD treated (n = 5; 3 males and 2 females) and untreated COVID-19 patients (n = 4; 2 males and 2 females) were randomly chosen from cohorts of VitD treated (n = 43; 36 males and 7 females) or untreated (n = 37; 27 males and 10 females) COVID-19 patients. Statistic test: comparison was done using unpaired t-test or Mann–Whitney U test, depending on the skewness of the data. *P < 0.05, **P < 0.01.

Similar to what we observed in whole blood of these patients, mRNA and protein levels of VDR were elevated in the saliva samples of COVID-19 patients following VitD treatment (Fig. 3A–C, 0.58 log FC increase of VDR mRNA, P < 0.0001). Furthermore, an increase in RIG-1/MDA-5 signaling pathway, measured by mRNA and/or protein levels of RIG-1, MDA-5, and p-IRF3 (Fig. 3D–J, around 2 FC increase of RIG-1, MDA-5, and IRF3 mRNA levels, P < 0.0001), and higher activity of JAK-STAT pathway, measured by levels of p-STAT1, p-STAT2, and p-JAK1 was observed in saliva of VitD treated compared to untreated COVID-19 patients (Fig. 3K–P, P = 0.001; Supplementary Fig. S3). As a consequent to activation of JAK-STAT pathway, the mRNA and protein levels of ISGs such as MX-1 and ISG-15 were also elevated in saliva of VitD treated COVID-19 patients compared to the controls (Fig. 3Q–V, 1.40 log FC increase of MX-1 mRNA, P < 0.0001; and 6.55 log FC increase of ISG-15 mRNA, P < 0.0001).

### VitD treated COVID-19 patients had lower all-cause in hospital mortality

**Figure 4:**
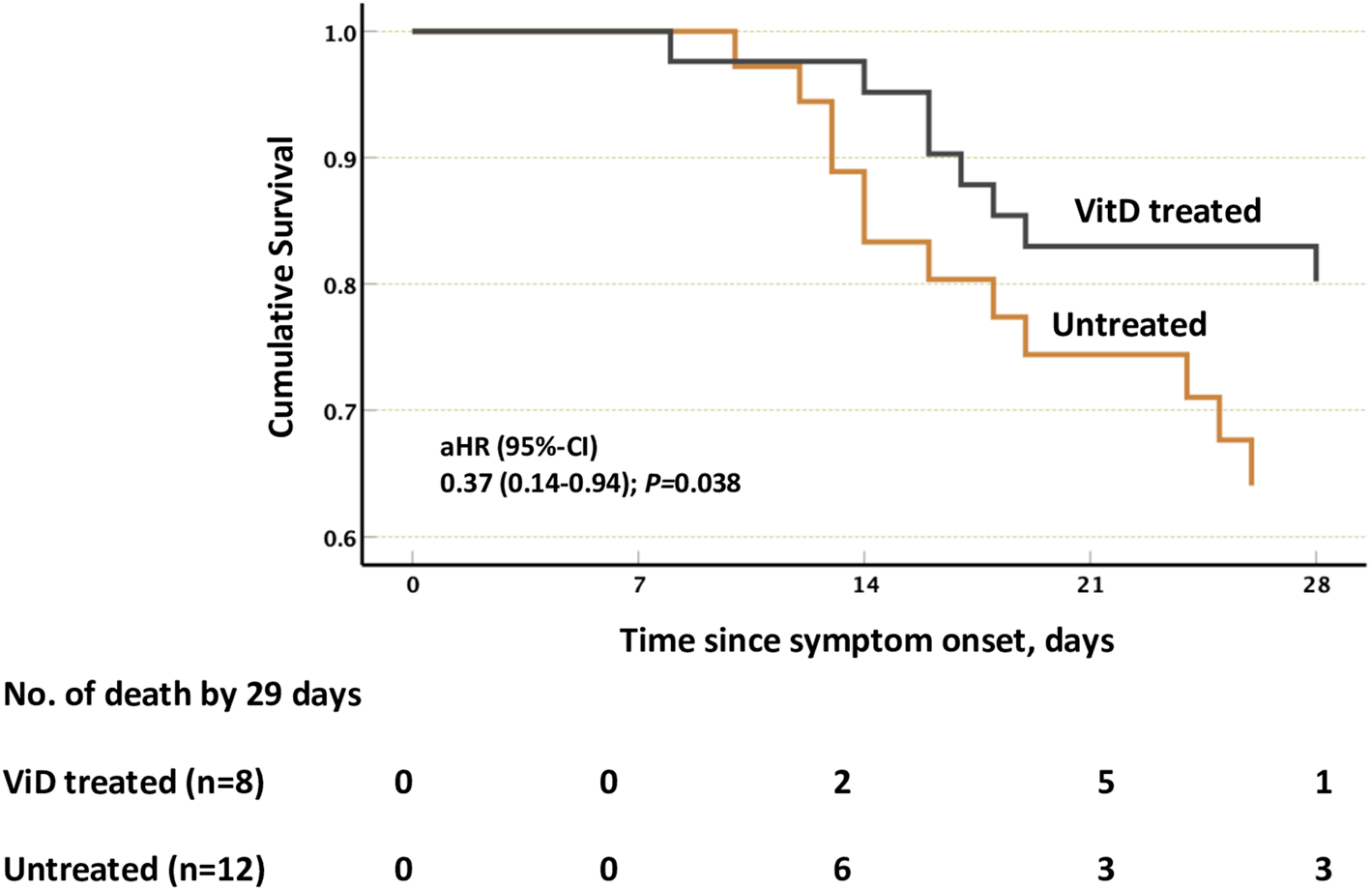
Lower 29-day all-cause mortality of VitD treated COVID-19 patients. The Kaplan–Meier curve of VitD treated (n = 43, 8 death events) and untreated COVID-19 patients (n = 37, 12 death events). The Cox proportional hazards regression model was adjusted for patient’s demographics factors (age, gender, and body mass index), comorbidities (diabetes mellitus), and COVID-19 related severity serum marker (D-dimer and C-reactive protein).

The effect of VitD treatment on all-cause mortality of COVID-19 patients was then evaluated. By day 29, 8 out of 43 patients (19%) in the VitD treated group died as compared to 12 out of 37 patients (32%) in the untreated groups (Fig. 4; adjusted hazard ratio, 0.37, 95% confidence interval of 0.14–0.94; P = 0.038). This result suggests that VitD treatment could contribute to improved immune responses and overall lower mortality most likely through enhancement of type I IFN signaling.

## Discussion

**Figure 5:**
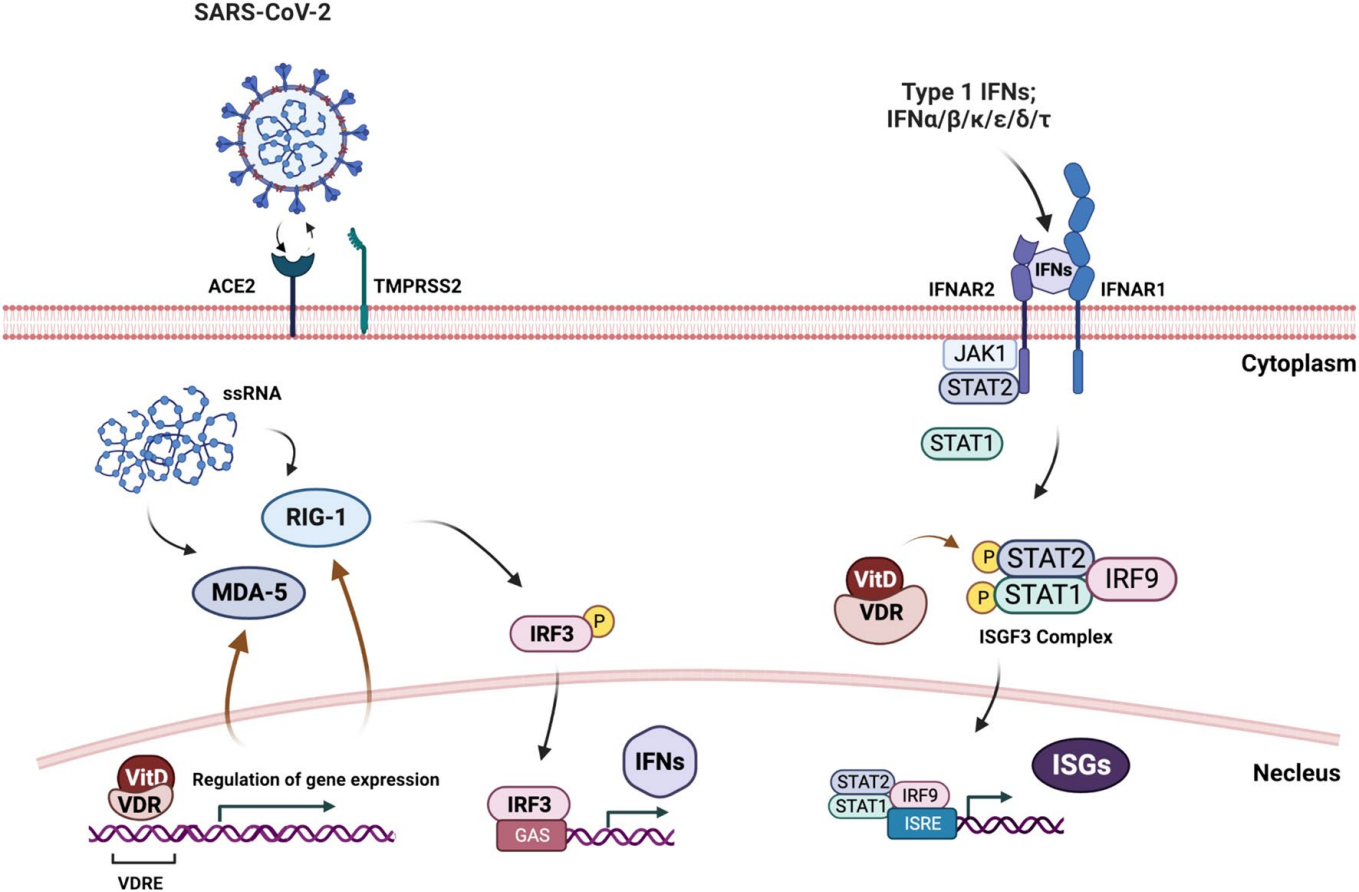
Schematic overview of the study findings. VitD enhances signaling of RIG-1/MDA-5 and JAK-STAT pathways and the resultant production of MX-1 and ISG-15 antiviral ISGs.

In this study we have demonstrated that VitD's anti-viral mechanisms can be linked to its ability to increase host IFN-α/β signaling both in vitro and in clinical settings among COVID-19 hospitalized patients by augmenting the signaling of RIG-1/MDA-5 and JAK-STAT pathways and the resultant MX-1 and ISG-15 antiviral ISGs (Fig. 5). This elevated IFN α/β signaling in conjunction with the ISGs, could then potentiate the innate immune responses to clear SARS-CoV-2 viral infections.

Following infection, several SARS-CoV-2 proteins, N, NSP16, and NSP16 inhibit RIG-1 and MDA-5 and the activation of downstream interferon regulatory factors (IRF3 or IRF7) needed for the production of IFN-α/β cytokines^39^. Interestingly, here we noticed an upregulation of RIG-1, MDA5 and IRF3 with VitD treatment in vitro, and in whole blood and saliva of VitD treated COVID-19 hospitalized patients. In line of these findings, Jadhav et al.^40^ have shown that VitD (Calcitriol; 1,25(OH)2D) treatment upregulated gene expression levels of RIG-1 in vitro in dengue virus infected macrophages. This ability of VitD to boost IFN signaling could be fundamental in the process of contracting the SARS-CoV-2 infection, as it was shown that SARS-CoV-2 manipulates the host’s innate immunity by dysregulating type I IFN immune responses^10^.

SARS-CoV-2 mediated inhibition of STAT1, and the subsequent shift to a STAT3-dominant signaling may lead to the proinflammatory conditions most commonly observed in hospitalized COVID-19 patients^39^. VitD treatment enhanced activation of JAK-STAT pathway in vitro, and in whole blood and saliva of treated COVID-19 hospitalized patients. The activation of JAK-STAT pathway by IFN-α/β leads to the upregulation of hundreds of ISGs for which, many have the ability to rapidly antagonize viruses within infected cells.

Moreover, IRF9, a key transcriptional factor involved in the JAK-STAT signaling pathway, and ISGs such as MX-1 and ISG-15 were upregulated following VitD treatment. IRF9 deficiency has been reported to increase susceptibility to several forms of viral infections and result in decreased levels of MX-1 and ISG-15^41^. MX-1 is a GTPase that is part of the antiviral response induced by IFN-α/β signaling. It was shown to inhibit influenza virus infection by blocking viral transcription and replication^42^. ISG-15 which is another important component of host response to viral infections, interferes with viruses’ localization, protease activity, or ability to interact with host proteins by binding to- or “ISGylation” of viral proteins^43^. Similarly, the expression of ISG-15 was enhanced following treatment of dengue virus infected macrophages with 1,25(OH)2D^40^.

IRF5 transcription factor, which was identified as one of the VitD target genes^37^, has recently been shown to be among the 22 transcription factors regulated by VitD, and that it also regulates transcription of other VitD target genes in monocytes^44^. In an in vitro experiment, VDR bound to the gene loci of IRF5 in VitD treated monocytes^44^. The report of VitD upregulating IRF5, which are downstream of TLR3, and are essential for type I signaling^45^, may provide further evidence behind the implication of VitD in TLR3/IFN signaling^46^. Further investigations of this effect of VitD is highly needed.

It is worth noting that the elevated IFN α/β signaling and level of ISGs, was apparent in both blood and the saliva of VitD treated COVID-19 patients. The rich blood circulation surrounding salivary gland has been shown to facilitate exchange of blood proteins into the saliva^47^. This indicates that saliva can be a reliable fluid for monitoring the blood level/activity of type I IFNs following treatment. Saliva has been studied as a potential diagnostic or prognostic tool in different disease and is expected to become a substitute for other biological fluids such as serum or urine^38,48^. Therefore, saliva can be a non-invasive source of biomarkers for monitoring COVID-19 severity and treatment.

Finally, the ability of VitD to elevate IFN α/β signaling could be beneficial for patients with COVID-19. It was reported that asymptomatic COVID-19 patients with elevated blood levels of type I IFNs and ISGs were associated with limited disease progression^49^. Moreover, VitD deficiency, defined as a blood level lower than 20 ng/mL (< 50 nmol/L), is likely be a significant factor behind COVID-19 severity and mortality^50^. In this regard, a randomized clinical trial of 76 patients hospitalized with COVID-19 in Spain found that treatment with high-dose VitD (oral calcifediol 0.532 mg, equal to oral 21,000 IU cholecalciferol) at admission significantly reduced the risk of intensive care unit admission^51^. An observational study of 324 patients with COVID-19 in Italy reported that VitD treatment (50,000 IU cholecalciferol per month) lowered risk of in-hospital death from COVID-19^52^. Here, higher dose of VitD was administrated to our studied cohort (50,000 IU cholecalciferol per week for 2–3 weeks) that has resulted in significantly lower COVID-19 related death by day 29. Our finding of the ability of VitD to increase type I IFN responses and the production of ISGs against SARS-CoV-2 highlights one of possible mechanisms by which VitD may improve immunity and hence survival of severe COVID-19 patients.

### Strengths and limitations

This study is the first to elucidate the ability of VitD to enhance the production and activity of type I IFN signaling pathway both in vitro, and in COVID-19 patients suffering from severe infection upon treatment. This ability of VitD to ameliorate IFN signaling could have important implications for SARS-CoV-2 viral infectivity since the virus manipulates the innate immunity by dysregulating type I IFN immune responses. Despite the strength of our study, there are few limitations that should be acknowledged. Firstly, all patients were followed up for 29 days only after their hospital admission which may limit report of events occurring after this period. Secondly, baseline serum levels of 25(OH)D concentrations were not available for patients. However, patents randomization to be treated or not with VitD was based on national guideline for COVID-19 treatment, and based on physician decision^25^. Lastly, VitD metabolism may be influenced by genetic factors, but this was not the focus of our study.

## Conclusion

In conclusion, our data showed that VitD could augment signaling of RIG-1/MDA-5 and IFN α/β pathways and the production of ISGs. Such findings suggest important connotations that VitD supplementation could help reduce the severity of COVID-19 disease by boosting innate immunity of patients and support the public health message for maintaining adequate VitD for the prevention and probably treatment of SARS-CoV-2 infection. Further studies and RCTs are needed to confirm the modulatory role of VitD on type I IFNs and decipher the efficacy and mechanism of VitD as a therapeutic agent in COVID-19 infection.

## Supplementary Information

Supplementary Information 1.

Supplementary Information 2.

Supplementary Information 3.

Supplementary Information 4.

Supplementary Information 5.

Supplementary Information 6.

## Data Availability

The datasets used and/or analyzed during the current study available from the corresponding author on reasonable request.
