# Supplementary material for: Vitamin D enhances type I IFN signaling in COVID-19 patients
Source: Sci Rep. 2022 Oct 22;12:17778. doi: 10.1038/s41598-022-22307-9 (PMC9588043; doi:10.1038/s41598-022-22307-9)
Supplement: Supplementary file 1 — Supplementary Information 1. [file 41598_2022_22307_MOESM1_ESM.pdf]

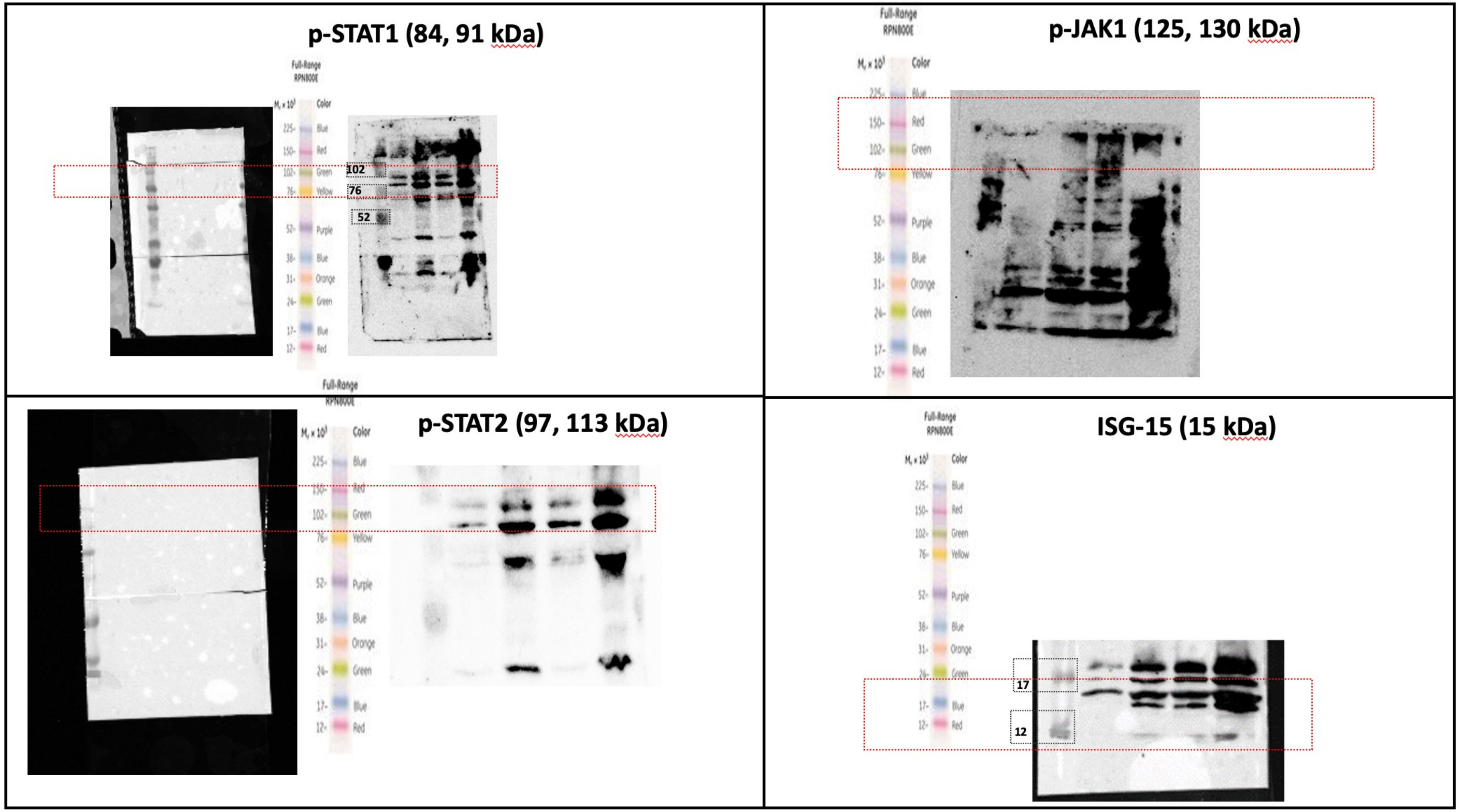

Supplementary Figure 1. Protein levels of p-STAT1, p-STAT2, p-JAK1, and ISG-15 in VitD and/or IFNα (50nM of calcitriol and/or 1μg/ml of IFNα for 8 hrs) treated PBMCs. (Figure 1F, 1H, and 1J). Blots were visualized on a BioRad ChemiDoc™ Touch Imager; p-STAT1, p-STAT2, and p-JAK1 exposure time of 1 to 2 minutes (signal accumulation), and ISG-15 exposure time of 30 seconds (Signal accumulation).
