# Supplementary material for: Vitamin D enhances type I IFN signaling in COVID-19 patients
Source: Sci Rep. 2022 Oct 22;12:17778. doi: 10.1038/s41598-022-22307-9 (PMC9588043; doi:10.1038/s41598-022-22307-9)
Supplement: Supplementary file 2 — Supplementary Information 2. [file 41598_2022_22307_MOESM2_ESM.pdf]

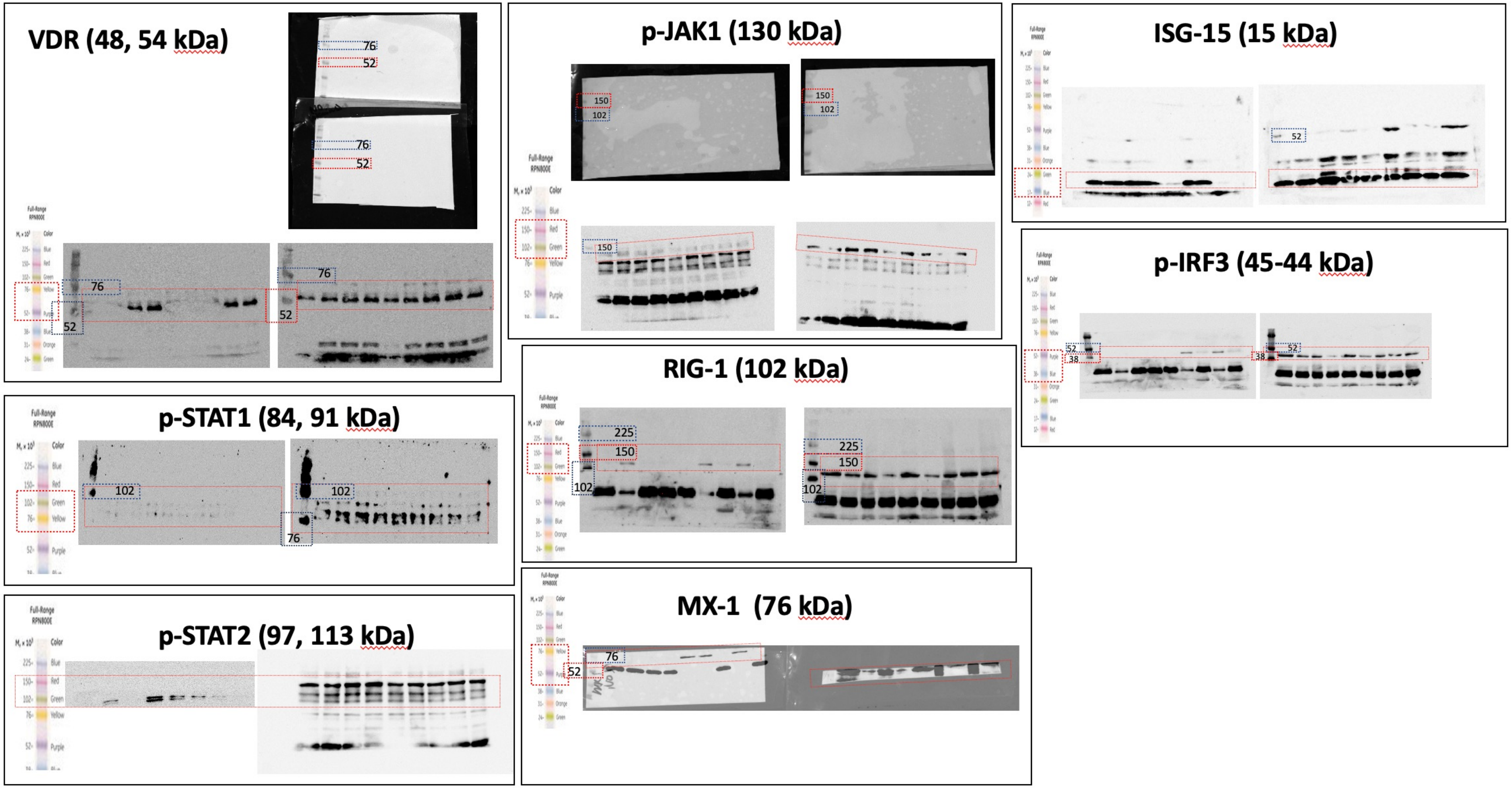


**Supplementary Figure 2.** Protein levels of VDR, p-STAT1, p-STAT2, p-JAK1, RIG-1, MX-1, ISG-15, and p-IRF3 in in whole blood of VitD treated (cholecalciferol was administrated as 50,000 IU weekly) and untreated COVID-19 patients. (Figure 2). Blots were visualized on a BioRad ChemiDoc™ Touch Imager; p-STAT1, p-STAT2, p-JAK1, and p-IRF3 exposure time of 1 to 2 minutes (signal accumulation), and VDR, RIG-1, MX-1, and ISG-15 exposure time of 10 to 30 seconds (Signal accumulation).
