# Supplementary material for: Vitamin D enhances type I IFN signaling in COVID-19 patients
Source: Sci Rep. 2022 Oct 22;12:17778. doi: 10.1038/s41598-022-22307-9 (PMC9588043; doi:10.1038/s41598-022-22307-9)
Supplement: Supplementary file 4 — Supplementary Information 4. [file 41598_2022_22307_MOESM4_ESM.pdf]

Supplementary Table 1. Gene expression datasets used in this study.

GEO accession	Platform	Sample	Condition 1	Condition 2
GSE106885	GPL18573	Normal human bronchial epithelium	100 nM calcitriol treated cells (n=3)	Untreated cells (n=3)
GSE106885	GPL18573	Normal human bronchial epithelium	10 μg/ml poly I:C treated cells (n=3)	Untreated cells (n=3)
GSE148829	GPL18573	Bronchial epithelial cells (BEAS2B)	10 ng/mL treated cells (n=3)	Untreated cells (n=3)
GSE147507	GPL23126	Normal human bronchial epithelial cells	SARS-Cov-2 infected cells (n=3)	Mock (n=3)
