# Supplementary material for: Vitamin D enhances type I IFN signaling in COVID-19 patients
Source: Sci Rep. 2022 Oct 22;12:17778. doi: 10.1038/s41598-022-22307-9 (PMC9588043; doi:10.1038/s41598-022-22307-9)
Supplement: Supplementary file 5 — Supplementary Information 5. [file 41598_2022_22307_MOESM5_ESM.pdf]

Supplementary Table 2. List of primer sequences used in qRT-PCR

Genes	Forward primer sequence (5′-3′)	Reverse primer sequence (5′-3′)
**RIG-1 (DDX58)**	TCCGGAAGACCCTGGACCCTAC	AAGTGTGGCAGCCTCCATTGG
**MX-1**	GCAAGGTCAGTTACCAGGACTACG	TGATTCCCATTCCTTCCCCGGC
**IRF9**	TGCTGCCACCAGGAATCGTCTC	GTCCTGGAGCACAGAGGGACTG
**IRF3**	TCTGCCCTCAACGCAAAGAAG	TACTGCCTCCATTGGTGTC
**MDA-5 (IFIH1)**	GGGAGTGGAAAAACCAGAGTGG	GCGGAAGAGCTGTTCAACTAGC
**ISG-15**	TGGGACCTGACGGTGAAGATGC	GCACGCCGATCTTCTGGGTGAT
**18s**	TGACTCAACACGGGAAACC	TCGCTCCACCAACTAAGAAC
